# Recognizing obsessive-compulsive disorder: how suitable is the German Zohar-Fineberg obsessive-compulsive screen?

**DOI:** 10.1186/s12888-021-03458-x

**Published:** 2021-09-11

**Authors:** Franziska Kühne, Tatjana Paunov, Florian Weck

**Affiliations:** Department of Psychology, Clinical Psychology and Psychotherapy, University of Potsdam, Karl-Liebknecht-Str. 24-25, 14476 Potsdam, Germany

**Keywords:** Assessment, Obsessive-compulsive disorder, Psychodiagnostics, Psychometric properties, Questionnaire, Screening

## Abstract

**Background:**

Despite the prevalence of obsessive-compulsive disorder (OCD), its precise identification remains challenging. With the Zohar-Fineberg Obsessive-Compulsive Screen (ZF-OCS; 5 or 6 items), a brief instrument is widely available mainly in English. As there is a lack of empirical studies on the ZF-OCS, the aim of the present study was to translate the items into German and investigate the instrument in a nonclinical sample.

**Methods:**

In two consecutive online surveys, *n* = 304 and *n* = 51 students participated. Besides the ZF-OCS, they answered established measures on OCD, depression, health anxiety, general anxiety and health-related well-being.

**Results:**

Whereas internal consistency was low (*α* = .53–.72; ω = .55–.69), retest reliability (*r*_t1,t2_ = .89) at two weeks was high. As expected, we found high correlations with other OCD instruments (*r* > .61; convergent validity), and significantly weaker correlations with measures of depression (*r* = .39), health anxiety (*r* = .29), and health-related well-being (*r* = −.28, divergent validity). Nonetheless, the correlations with general anxiety were somewhere in between (*r* = .52).

**Conclusions:**

Due to heterogeneous OCD subtypes, the ZF-OCS asks diverse questions which probably resulted in the present internal consistency. Nevertheless, the results on retest reliability and validity were promising. As for other OCD instruments, divergent validity regarding general anxiety seems problematic to establish. Even so, the ZF-OCS seems valuable for screening purposes, as it is short and easy to administer, and may facilitate initiating subsequent clinical assessment. Further studies should determine the instrument’s diagnostic accuracy.

**Supplementary Information:**

The online version contains supplementary material available at 10.1186/s12888-021-03458-x.

## Background

Obsessive-compulsive disorder (OCD) is characterized either by the presence of recurrent and persistent thoughts, urges or images perceived as intrusive and unwanted (*obsessions*) and/or by repetitive behaviors or mental acts that the individual feels driven to perform in response (*compulsions)* [1]. Obsessions and compulsions may refer to the symptom dimensions of a) contamination, b) responsibility for harm, injury, or bad luck, c) symmetry, completeness, or exactness or d) violent, sexual or religious thoughts [2]. OCD is a prevalent disorder with a 12-month prevalence ranging between 1.2% in the US American [3] and 3.6% in the German general population [4]. OCD substantially and negatively affects quality of life [5], social life, work and family life [6].

Despite the impairment associated with OCD, many years may pass until individuals receive adequate treatment [7]. For example, Wahl and colleagues [8] determined that only 28% of outpatients diagnosed with OCD by the study team had received the correct diagnosis during a previous visit to their psychiatric practice. Not only in specialized settings, but also in primary care, under-recognition represents a considerable challenge. According to recent meta-analyses, the mean sensitivity of general practitioners to correctly diagnose anxiety and other mental disorders accounts for around 42–50% only [9]. This is highly relevant for OCD, as patients may present their symptoms to various physicians (e.g., due to fear of cancer or an infectious disease) or may overemphasize physical symptoms such as dermatitis resulting from excessive washing [10]. OCD patients may also withhold symptoms (such as religious or aggressive obsessions) because of shame, a prominent emotion in OCD [11]. Furthermore, they may describe other complaints such as depressive symptoms, traumatic life events, or family or sleeping problems to their physician [12] which then misleads the doctor.

Since under-recognition presents a problem for timely allocation to adequate treatment, several authors recommend routinely using screening questions during any mental state examination in specialized and in general health care settings [8, 13]. Nevertheless, there is a lack of validated, brief OCD screening tools [14], especially in Germany [7]. One instrument that was proposed for active screening purposes is the Zohar-Fineberg Obsessive Compulsive Screen (ZF-OCS; [13]). It comprises the following five questions that take only a few minutes to go through: 1) “Do you wash or clean a lot?”, 2) “Do you check things a lot?”, 3) “Is there any thought that keeps bothering you that you’d like to get rid of but can’t?”, 4) “Do your daily activities take a long time to finish?”, and 5) “Are you concerned … either … about orderliness and symmetry [10, 13] or … about putting things in a special order or are you very upset by mess?” [14].

The ZF-OCS was validated in dermatology outpatients against the OCD item of the Mini-International Neuropsychiatric Interview [15], and it was found to have excellent sensitivity (94%) and good specificity (85%; [16]). Therefore, it was included in national guidelines and considered a promising screening tool [7, 14]. Although the German Association for Psychiatry, Psychotherapy and Psychosomatics [7] offers a translation, no validation study has been published so far. To the best of our knowledge, the only available results were briefly published from a pilot study, and indicate excellent sensitivity and specificity, as well as good internal consistency of the tool [8]. In addition to the five ZF-OCS items, the authors of the pilot study used additional questions for the assessment of symptom severity and impairment, which however, they did not publish [8]. Accordingly, using additional questions was proposed by the national guideline as well [7], without specification of which questions to use.

The purpose of the current study was to investigate the reliability and validity of the German ZF-OCS in a non-clinical sample. We hypothesized that the ZF-OCS would show acceptable internal consistency as well as good retest reliability. We further expected evidence of convergent validity, i.e., high correlations with other OCD measures, and evidence of discriminant validity defined by weaker correlations with measures of anxiety, depression, and health-related well-being. Furthermore, we explored the psychometric properties of the ZF-OCS with and without an additional question on the individual impairment caused by current OC symptoms.

## Methods

### Participants and procedure

Participants were recruited at the University of Potsdam from July 2019 to January 2020 (main sample, before the Corona pandemic spread exponentially in Germany) and from June 2020 to August 2020 (independent retest sample with a 2 weeks retest interval, timeframe with reduced Corona infections in Germany). Individuals took part in the online survey voluntarily, gave informed consent, and received course credit for participation. Besides being an adult, there were no prerequisites for study participation. The study was approved by the Ethics review board of the University of Potsdam (no. 2/2019, no. 51/2019, no. 22/2020). The complete data set was used for validation of another OCD screening tool as well [17].

### Instruments

#### Zohar-Fineberg obsessive compulsive screen (ZF-OCS; (7, 13))

We used the translation of the 5-item ZF-OCS published by the German Association for Psychiatry, Psychotherapy and Psychosomatics [7]. The screening is deemed positive if a person answers at least one of the five ZF-OCS items positively. In the retest sample, we decided to add an item on individual impairment. As proposed by two previous publications [10, 14], we decided to add the item “Do these problems trouble you?” (in German “Beeinträchtigen Sie diese Probleme?”). In this case, the screening is deemed positive if a person answers at least one of the five ZF-OCS items positively, and if he or she perceives being impaired [7]. Therefore, sum scores ranged between 0 and 5 resp. 6.

#### Dimensional obsessive-compulsive scale (DOCS; (2, 18))

The DOCS covers the four main symptom dimensions of OCD: contamination, responsibility for harm, symmetry and thoughts that are perceived as unacceptable. Regarding each dimension, participants also indicate severity (i.e., time expenditure, avoidance behavior, distress, functional interference and difficulties in refraining). The items are rated on a 5-point Likert scale from zero (*no symptoms*) to four (*extreme symptoms*). Factor analysis revealed a stable four-factor structure complemented by a general OCD factor [2]. Evidence of stability, construct validity and diagnostic accuracy was provided as well [2, 19]. The German translation displayed excellent reliability (α = .91), satisfactory to good construct validity and satisfactory diagnostic accuracy [18]. In the current study, the DOCS showed excellent internal consistency (α = .91).

#### Obsessive-compulsive inventory-revised (OCI-R; (20, 21))

The OCI-R is an 18-item questionnaire on common OCD symptoms. Its items are rated on a 5-point Likert scale from zero (*not at all*) to four (*very much*). The OCI-R comprises six subscales on washing, checking, ordering, obsessing, neutralizing and hoarding. Research supported its six-factorial structure in a German clinical sample [21]. Foa and colleagues [20] reported excellent test-retest reliability. Strong support was found for its divergent [21] and convergent validity [22]. In the present study, it showed good internal consistency (α = .88).

#### Patient health Questionnaire-2 (PHQ-2; (23))

The PHQ-2 is a brief screening tool for symptoms of depression. Both items are rated on a 4-point Likert scale ranging from zero (*not at all*) to three (*nearly every day*). Evidence of its construct validity was provided by an association with risk factors of depression in the general population [23]. In the present study, the 2-item PHQ-2 yielded acceptable internal consistency (α = .78).

#### Generalized anxiety disorder Scale-7 (GAD-7; (24, 25))

The GAD-7 screens for anxiety symptoms. Its items are rated on a 4-point scale ranging from zero (*not at all*) to three (*nearly every day*). There is evidence of a one-dimensional factor structure, for its construct validity [25, 26] and for diagnostic accuracy [24]. In the current study, the GAD-7 showed good internal consistency (α = .85).

#### Illness attitude scale-bodily preoccupation subscale (IAS-BP; (27))

The IAS-BP is the 3-item subscale of the IAS on bodily preoccupations. The items are rated on a 5-point Likert scale ranging from zero (*never*) to four (*mostly*). Weck et al. [27] described the IAS-BP as a valid screening instrument for health anxiety, and provided evidence of its diagnostic accuracy, for convergent and for divergent validity [27, 28]. In the present study, the IAS-BP showed acceptable internal consistency (α = .78).

#### World Health Organization well-being index (WHO-5; (29))

The WHO-5 is a short measure on subjective well-being. It comprises five items rated on a 6-point scale from zero (*at no time*) to five (*all of the time*). Raw sum scores were converted to percentages by multiplying them by four. Brähler et al. [29] provided evidence of the instrument’s reliability and construct validity. In the present study, the WHO-5 showed good internal consistency (α = .85).

### Data analysis

Statistical analyses were performed using IBM SPSS Statistics 24, the level of significance was set at .05 for all analyses. If there were single missing values, they were replaced by individual means.

#### Reliability

As it is the more established statistic, we investigated Cronbach’s α first. According to Bland and Altman [30], Cronbach’s α should be interpreted as follows: .70–.80 acceptable, >.80 good, and > .90 excellent. Since Cronbach’s α applies more to items that clearly measure the same construct, and the ZF-OCS refers to various OCD symptom dimensions, McDonald’s ω constitutes an alternative statistic in our study [31]. For its calculation, we used the OMEGA macro and syntax (31). If McDonald’s ω is >.80, it may be interpreted as good [32]. Furthermore, we calculated the corrected item-total correlations, whereby values >.40 may be interpreted as good [33].

Concerning retest reliability at 2 weeks, Pearson correlations and paired t-tests were calculated for the ZF-OCS sum scores between both measurement times. Correlations were considered high if *r* was ≥.50 [34].

#### Convergent and divergent validity

Pearson correlations were computed again, and considered high if they were ≥ .50 [34]. To investigate whether correlations between the ZF-OCS and the convergent measures were significantly stronger than the correlations between the ZF-OCS and the divergent measures, *t*-tests for comparing dependent correlations were used [35].

## Results

Participants in the main sample (*N* = 304) were on average 24.86 years old (*SD* = 6.93). They were mostly female (76.3%, *n* = 232) students (85.5%, *n* = 260). Being asked for prior diagnoses given by a physician or psychologist, seven participants (2.3%) self-reported having been diagnosed with OCD, and *n* = 52 (17.1%) stated having been diagnosed with depression before. Concerning the ZF-OCS, 28.9% (*n* = 88) of the participants did not answer positively to any item, 28.9% (*n* = 88) affirmed one item, 18.1% (*n* = 55) affirmed two items, 15.1% (*n* = 46) three items, 6.9% (*n* = 21) four, and 2% (*n* = 6) affirmed all five items (for means and standard deviations of all measures, see Table 1).

**Table 1:** Means and Standard Deviations in the Main Sample

	*M*	*SD*	*Range*
ZF-OCS	1.48	1.34	0–5
OCI-R	14.06	9.84	0–45
DOCS	10.91	9.29	0–42
PHQ-2	1.74	1.40	0–6
GAD-7	5.79	4.12	0–20
IAS-BP	3.54	2.38	0–12
WHO-5	49.77	18.76	8–96

The independent retest sample comprised *N* = 51 adults with an average age of *M* = 25.67 years (*SD* = 5.29). 78.4% were female (*n* = 40), and most of them were students (78.4%, *n* = 40). There were no significant differences between the main and the retest samples regarding age and gender (*p* > .05).

Whereas between 21.7 and 38.8% of the participants responded positively to at least one of the five OC items (main sample, see Table 2), 15.7% (*n* = 8, retest 1) resp. 13.7% (*n* = 7, retest 2) confirmed the impairment item.

**Table 2:** Intercorrelations between the ZF-OCS items in the Main (*n* = 304) and Retest (*n* = 51) Samples

Main Sample	Item 1 *wash*	Item 2 *check*	Item 3 *thought*	Item 4 *time*	Item 5 *order*	Item-total^a^	Yes %
Item 1	1	.270**	.179**	.073	.146*	.269	24.3
Item 2		1	.243**	.142*	.322**	.413	34.2
Item 3			1	.227**	.102	.297	28.9
Item 4				1	.137*	.227	21.7
Item 5					1	.286	38.8
**Retest** **Time 1**	Item 1 *wash*	Item 2 *check*	Item 3 *thought*	Item 4 *time*	Item 5 *order*	Item 6 *impairment*	
Item 1	1	.123	.142	.159	.312*	.142	
Item 2		1	.173	.056	.409**	.289*	
Item 3			1	.429**	.095	.852**	
Item 4				1	.165	.560**	
Item 5					1	.206	
Item 6						1	
**Retest** **Time 2**	Item 1 *wash*	Item 2 *check*	Item 3 *thought*	Item 4 *time*	Item 5 *order*	Item 6 *impairment*	
Item 1	1	.289*	.104	.240	.336*	.206	
Item 2		1	.432**	.215	.334**	.368**	
Item 3			1	.485**	.065	.739**	
Item 4				1	.242	.551**	
Item 5					1	.245	
Item 6						1	

*Notes*. * *p* < .05, ** *p* < .01 (two-tailed)

^a^corrected item-total correlations

### Reliability

Cronbach’s *α* lay between .53 (main sample), and .72 (retest sample t_2_, 6 items, see Table 3), and only the latter value may be interpreted as acceptable [35]. McDonald’s ω lay between .55 (main sample) and .69 (retest sample t_2_, 6 items). The corrected item-total correlations were good (i.e., above .40) for item 2 on checking only (see Table 2).

**Table 3:** Reliability as Measured by Cronbach’s α and McDonald’s ω

	*α*	*ω*
Main sample	.53	.55
Retest sample t_1_, 5 items	.57	.58
Retest sample t_1_, 6 items	.68	.66
Retest sample t_2_, 5 items	.65	.64
Retest sample t_2_, 6 items	.72	.69

Regarding retest reliability at 2 weeks, the ZF-OCS sum scores (5 items) did not change significantly (*M*_t1_ = 1.31, *SD*_t1_ = 1.32; *M*_t2_ = 1.29, *SD*_t2_ = 1.39; *t* (50).227, *p* = .821). The Pearson correlation was *r*_t1,t2_ = .898, indicating a high correlation.

Similarly, the ZF-OCS sum scores including the item on impairment (6 items) did not change significantly during the retest interval (*M*_t1_ = 1.47, *SD*_t1_ = 1.58; *M*_t2_ = 1.43, *SD*_t2_ = 1.63; *t* (50).375, *p* = .709). The Pearson correlation was *r*_t1,t2_ = .892, indicating a high correlation as well.

### Convergent and divergent validity

For the main sample, Pearson correlations between the German ZF-OCS and the convergent measures were high, i.e., *r* = .64 (DOCS), and *r* = .61 (OCI-R) respectively (for all correlations, see Table 4).

**Table 4:** Bivariate Pearson Correlations between all Measures in the Main Sample

	OCI-R	DOCS	PHQ-2	GAD-7	IAS-BP	WHO-5
ZF-OCS	.61**	.64**	.39**	.52**	.29**	−.28**
OCI-R		.75**	.39**	.57**	.42**	−.30**
DOCS			.44**	.66**	.39**	−.34**
PHQ-2				.65**	.11	−.66**
GAD-7					.32**	−.58**
IAS-BP						−.13*

*Notes*. * *p* < .05, ** *p* < .01 (two-tailed)

The Pearson correlations between the ZF-OCS and the divergent measures were lower, i.e., *r* = .39 (PHQ-2), *r* = .29 (IAS-BP), and *r* = −.28 (WHO-5). Nevertheless, the ZF-OCS and the GAD-7 were associated more strongly (*r* = .52).

Most correlations with convergent measures were significantly stronger than the correlations with the divergent measures (Supplement 1) which did not apply to the GAD-7. In that case, the correlation between the GAD-7 and the DOCS (*r* = .66) was similar, but significantly stronger than the correlation between the ZF-OCS and the DOCS (*r* = .64, *p* = .001).

## Discussion

The Zohar-Fineberg Obsessive-Compulsive Screen (ZF-OCS) is described as “one of the most useful sets of screening questions” ([10] , p. 7) for detecting obsessive-compulsive (OC) symptoms. Although it was adopted by clinical guidelines [7, 14], the American Psychiatric Association [36] recommends choosing from slightly different screening questions. At the same time, the APA [36] emphasizes the need for developing a psychometrically sound screening instrument for use in primary care, as one of the key goals in current OCD research. The present study is one of the few to investigate the psychometric properties of the ZF-OCS, aiming to provide general practitioners with empirical data on the usefulness of this brief scale.

First of all, we investigated the internal consistency of the instrument, which, regardless of which coefficient we used, was low. This result indicates that we were not able to measure one coherent construct. First, the ZF-OCS covers the most prevalent OC symptom dimensions [2], ranging from obsessional thoughts, to washing/cleaning and checking compulsions through to symmetry. Since the OC symptom dimensions are rather heterogeneous [2], good or even excellent internal consistency of such a short scale may not be expected [33]. Patients may be affected, for example, by contamination obsessions and cleaning compulsions, but not by repugnant obsessional thoughts and neutralizing strategies [2]. Nevertheless, a screening tool should cover all dimensions roughly. Furthermore, low internal consistency may also result from poor item interrelatedness [37]. Thus, we examined the corrected item-total correlations of the ZF-OCS, whereby it was good for one item only. Moreover, the ZF-OCS is a brief instrument, and its small number of items may contribute to its low internal consistency [33]. Since screening instruments should enable rough estimations with minimal effort, their reliability is often lower than that of more detailed measures [33].

Despite low internal consistency, retest reliability of a questionnaire may be high if the underlying construct is temporally stable [33]. As our second survey suggested, retest reliability was indeed high, both with and without including the additional item on individual impairment. Nevertheless, since we cannot exclude a memory effect [33], future studies should cover larger retest periods.

The high correlations of the ZF-OCS with established OCD measures (i.e., [18, 21]) suggests its convergent validity. As expected, the ZF-OCS screening also showed evidence of discriminant validity, since it was correlated significantly less with short measures on depression [23], health anxiety [27] and health-related well-being [29]. However, it turned out to be difficult to disentangle OC symptoms from general anxiety as measured by the GAD-7 – a problem that appeared in previous studies on OCD instruments as well [2, 17, 18, 38]. The reason for this may be twofold. First, OCD is conceptually related to the anxiety disorders [39]. Second, the GAD-7 asks rather broad questions on anxiety and general distress (such as “Feeling afraid as if something awful might happen”) which may be easily confused with OC symptoms.

We further examined the effect of an additional item on impairment. We included the item in our retest survey, whereby the additional item increased internal consistency as described above. Although between 22 and 39% of the participants approved at least one of the first five ZF-OCS items, this was true for maximally 16% regarding perceived impairment. This supports the suggestion to assess the screening as positive only if a participant approved at least one of the five ZF-OCS items *and* perceives impairment [7] – a suggestion that also reflects the current diagnostic criteria [1].

The Institute for Quality and Efficiency in Health Care [40] defines “screening” as the examination of symptom-free (mostly healthy) persons for early recognition of diseases. Although Fineberg and colleagues [16] report excellent sensitivity and good specificity of the ZF-OCS in a dermatological sample, the German guideline authors indicate lower specificity due to their clinical experience with the scale [7]. Nevertheless, underrecognition is associated with undertreatment of OCD [12]. Therefore, false-positives may be acceptable here, as screening aims at initiating more detailed diagnostic procedures, referral to specialized treatment, thus counteracting possible chronification of the disease [7, 33]. Nonetheless, future studies with patient groups should investigate the sensitivity and specificity of the ZF-OCS, using an established diagnostic interview on mental disorders. Furthermore, further research could investigate the ZF-OCS as a self-report vs. a clinician-administered instrument, thus providing information on the most useful way to implement the scale.

## Conclusions

The present study investigated the psychometric properties of the ZF-OCS as a screening tool for OC symptoms. Although internal consistency was improvable, retest reliability, and convergent and divergent validity were very good. Given that the ZF-OCS includes five items, and one item on individual impairment only, we regard it as a feasible measure for use in basic health care settings. Our results suggest that including the impairment item is useful. However, we suggest further investigation of the instrument’s diagnostic accuracy in different samples (e.g., in specialized mental health care).

## Supplementary Information

**Additional file 1 Supplement 1.** T-tests for Comparing Dependent Correlation Coefficients.

## Data Availability

The datasets used and/or analyzed during the current study are available from the corresponding author on reasonable request.

## Abbreviations

APA: American Psychiatric Association
DGPPN: German Association for Psychiatry, Psychotherapy and Psychosomatics
DOCS: Dimensional Obsessive-Compulsive Scale
GAD-7: Generalized Anxiety Disorder Scale-7
IAS-BP: Illness Attitude Scale-Bodily Preoccupation Subscale
OCD: Obsessive-compulsive disorders
OCI-R: Obsessive-Compulsive Inventory-Revised
PHQ-2: Patient Health Questionnaire-2
WHO-5: World Health Organization Well-Being Index
ZF-OCS: Zohar-Fineberg Obsessive-Compulsive Screen
