# Correction to: Recognizing obsessive-compulsive disorder: how suitable is the German Zohar-Fineberg obsessive-compulsive screen?

**DOI:** 10.1186/s12888-022-03692-x

**Published:** 2022-01-21

**Authors:** Franziska Kühne, Tatjana Paunov, Florian Weck

**Affiliations:** Department of Psychology, Clinical Psychology and Psychotherapy, University of Potsdam, Karl-Liebknecht-Str. 24-25, 14476 Potsdam, Germany

Following the publication of the original article [1], the authors identified that the funding note was incorrect. The correct funding note is given below.

Funding

This research did not receive any specific grant from funding agencies in the public, commercial, or not-for-profit sectors. We acknowledge the support of the Deutsche Forschungsgemeinschaft and Open Access Publishing Fund of University of Potsdam”.

The original article [1] has been corrected.
