# Supplementary material for: Recognizing obsessive-compulsive disorder: how suitable is the German Zohar-Fineberg obsessive-compulsive screen?
Source: BMC Psychiatry. 2021 Sep 11;21:450. doi: 10.1186/s12888-021-03458-x (PMC8436546; doi:10.1186/s12888-021-03458-x)
Supplement: Supplementary file 1 — Additional file 1 Supplement 1. T-tests for Comparing Dependent Correlation Coefficients. [file 12888_2021_3458_MOESM1_ESM.docx]

**Supplement 1** *T-tests for Comparing Dependent Correlation Coefficients.*

|  | Compared measures | *r_xy_* | *r_xz_* | *r_yz_* | *t* | *df* | *p* |
| --- | --- | --- | --- | --- | --- | --- | --- |
| 1 | *r_xy_*=ZF-OCS/OCI-R  *r_xz_*=ZF-OCS/PHQ-2  *r_yz_*=OCI-R/PHQ-2 | .61 | .39 | .39 | 4.340 | 301 | .000 |
| 2 | *r_xy_*=ZF-OCS/DOCS  *r_xz_*= ZF-OCS/PHQ-2  *r_yz_*=DOCS/PHQ-2 | .64 | .39 | .44 | 5.271 | 301 | .000 |
| 3 | *r_xy_*=ZF-OCS/OCI-R  *r_xz_*=ZF-OCS/GAD-7  *r_yz_*=OCI-R/GAD-7 | .61 | .52 | .57 | 2.169 | 301 | .031 |
| 4 | *r_xy_*=ZF-OCS/DOCS  *r_xz_*=ZF-OCS/GAD-7  *r_yz_*=DOCS/GAD-7 | .64 | .52 | .66 | 3.300 | 301 | .001 |
| 5 | *r_xy_*=ZF-OCS/OCI-R  *r_xz_*=ZF-OCS/IAS-BP  *r_yz_*=OCI-R/IAS-BP | .61 | .29 | .42 | 6.392 | 301 | .000 |
| 6 | *r_xy_*=ZF-OCS/DOCS  *r_xz_*=ZF-OCS/IAS-BP  *r_yz_*=DOCS/IAS-BP | .64 | .29 | .39 | 6.998 | 301 | .000 |
| 7 | *r_xy_*=ZF-OCS/OCI-R  *r_xz_*=ZF-OCS/WHO-5  *r_yz_*=OCI-R/WHO-5 | .61 | -.28 | -.30 | 11.876 | 301 | .000 |
| 8 | *r_xy_*=ZF-OCS/DOCS  *r_xz_*=ZF-OCS/WHO-5  *r_yz_*=DOCS/WHO-5 | .64 | -.28 | -.34 | 12.286 | 301 | .000 |
